# The Relationship between Take-Off Parameters and Relative Vertical Momentum of Free Limbs at the Take-Off in Hurdle Clearance

**DOI:** 10.5114/jhk/176106

**Published:** 2024-05-17

**Authors:** Yusuke Ozaki, Takeshi Ueda

**Affiliations:** 1 Graduate School of Humanities and Social Sciences, Hiroshima University, Higashihiroshima City, Japan.

**Keywords:** athletics, obstacles, stiffness, free leg, coordination

## Abstract

This study aimed to clarify the kinetics of the relative vertical momentum to the proximal joint of each free limb and their contribution to the increase in the centre of mass height at the take-off of hurdle clearance, as well as their relationship with take-off variables. Thirteen male hurdlers cleared one hurdle at the height of their centre of mass, and their attempts were filmed using six high-speed cameras. The hurdle height was 96.54 ± 2.63 cm (55.35 ± 0.29% of body height). The approach distance was set at 15 m and adjusted by each hurdler in the range of 10–50 cm so as not to involve any noticeable step length adjustment before the take-off. The combined free limb relative vertical momentum tended to increase until mid-support and was maintained until the toe off. The smaller the whole-body vertical momentum at the toe off and the increase in relative vertical momentum of the lead leg during the take-off, the higher the take-off velocity, the shorter the support time, and the smaller the deceleration. The higher the relative vertical momentum of the forward swing arm during touchdown and the smaller the relative vertical momentum increase of the combined free limb and the forward swing arm during the take-off, the smaller the deceleration. In conclusion, hurdlers should reduce the increase in whole-body vertical momentum at the take-off by suppressing the increase in relative vertical momentum of the lead leg and the forward swing arm.

## Introduction

In track and field hurdle events (110 m, 100 m, or 400 m hurdles), sprinting time is greater than in sprint events without hurdles because of the duration of the hurdle clearance. Top hurdlers do not typically have good sprinting ability (Bedini, 2016; Iskra and Pietrzak, 2016). Hurdlers never get the chance to reach their maximum sprint speed as in sprint events without hurdles, thus having higher maximum sprint speeds is not the primary aim in training. Sprint hurdling, specifically the take-off of the hurdle step, has the greatest deceleration among all steps because it is accompanied by an increase in the body centre of mass (CM) height (Nagahara et al., 2021). However, high-level hurdlers maintain high sprinting speeds owing to the short take-off support time, short flight time and small deceleration (Čoh et al., 2020; González-Frutos et al., 2020; McDonald and Dapena, 1991b; Nagahara et al., 2021; Krzysztofik et al., 2023). Therefore, support time and deceleration in the hurdle clearance take-off are important variables. Determining the factors that minimize these variables is an important research objective to achieve high performance in hurdlers.

In the hurdle clearance take-off, the hurdler must gain the necessary whole-body vertical momentum at the toe off (WBVM_TO_) to clear a hurdle of a prescribed height. The main contributor to the achievement of vertical momentum in other jumping tasks, such as a vertical jump, a high jump, and running, is the stance leg, but the free limbs also contribute. The contribution of free limbs to WBVM_TO_ is determined by the positive increase in relative vertical momentum (RM) to the proximal joint of each free limb during the support phase (Lees and Barton, 1996; Lees et al., 2000). In analyses using the RM method, the combined free limb contribution to WBVM_TO_ was reported to be 7.1% (Lees et al., 2000), 12.7%, and 4.2% (Lees and Barton, 1996) for the high jump, the vertical jump, and running, respectively. Therefore, the contribution of free limbs to the increase in CM height at the take-off should also be observed in hurdle clearance. However, a hurdler should limit the increase in CM height to the minimum required to clear the hurdle. Thus, large WBVM_TO_ and the contribution of free limbs may be detrimental to the take-off of hurdle clearance. Additionally, hurdle clearance may be different from other jumping tasks in terms of specific free limb RM changes and their relationship with performance. Furthermore, the position of these free limbs and the swing velocity related to the RM of the free limbs during the support phase affect the take-off variable via the distance between the contact foot and the CM (Hunter et al., 2005), which is related to the deceleration and acceleration of the CM during the support phase. Therefore, the RM behaviour of these free limbs may be a determinant of the take-off variables (take-off velocity, support time, and deceleration) in hurdle clearance. However, no biomechanical studies in hurdle clearance have analysed the RM of free limbs. Determining factors contributing to hurdle clearance may provide new insights that can inform coaches and athletes, considering movements of the upper and lead legs.

This study aimed to clarify the kinetics of the RM of free limbs and their contribution to the increase in CM height at the take-off of hurdle clearance, as well as their relationship with the take-off variables (take-off velocity, velocity change rate, and support time). The hypothesis of this study was as follows: hurdlers with shorter take-off support times, smaller deceleration, and higher take-off velocity would have lower WBVM_TO_ and lower free limb contribution to WBVM_TO_.

## Methods

### Participants

Participants included 13 male college hurdlers (age, 20.54 ± 2.17 years; body height, 1.74 ± 0.04 m; body mass, 67.80 ± 4.30 kg) who had experience in either or both the 110 m and 400 m hurdle events; four and five participants had experience only in the 400 m and 110 m hurdles, respectively, while four participants had experience in both events. The personal records in each event were 17.16 ± 1.69 s (110 m hurdles) and 53.79 ± 2.48 s (400 m hurdles). The inclusion criteria were male college hurdlers with at least three years of hurdle running training and no history of injury in the past two months. The participants' CM horizontal velocity immediately after the take-off was 6.71 ± 0.51 m/s, as measured by the method described below. All participants were informed of the experimental procedures and risks, and provided written informed consent prior to participation in the study. The experiment was conducted without discomfort to the participants, in accordance with the Declaration of Helsinki. The ethics review board of the Graduate School of Humanities and Social Sciences, Hiroshima University approved the experimental protocol (approval number: 2021062; approval date: 3 August 2021).

### Measures

The experiments were conducted on an outdoor synthetic track. Participants had to clear the first hurdle that was set up at the 15^th^ m from the start at the fastest possible speed. The hurdle height was 96.54 ± 2.63 cm (55.35 ± 0.29% of body height, see below), adapted to CM height at the anatomical upright position of each participant. This height was equivalent to a relative hurdle height of 57.1% for world-class male hurdlers (Hanley et al., 2021). In this study, the hurdle posts were fixed with tape so that they could be set at any height. Hurdle height was measured using a measurement pole with a leveller. The experiment was conducted using a standing start, with participants wearing spiked shoes. Thirty coloured markers were attached to each participant’s body and shoes to ensure that body feature points could be identified. The marker positions were the top of the head, the upper margin of the sternum, the right and the left tragion, the acromion, greater trochanters, medial and lateral epicondyles, the styloid process of the radius and ulnar, the head of the third metacarpal bone, the medial and the lateral epicondyle of the femur, medial and lateral malleolus, the heel (on shoes), and the toe (on shoes).

Before the experiment, CM height of each participant was determined. Each participant was placed upright at the centre of a square made of ground markers placed at 2.5-m intervals, and their entire body was filmed with a high-speed camera from the front. A 2.05-m pole was used to calibrate the aspect ratio for vertical and horizontal measurements to precalculate the CM height of participants needed to set individual hurdle heights.

To construct a three-dimensional (3D) coordinate system from before the take-off to after landing, we set up a filming area of 6.0 m in the running direction, 1.2 m in lane width, and 2.0 m in height, centered on the hurdle position. Six fixed high-speed cameras (512 × 384 pixels, 240 frames/s, F5.0, and a shutter speed of 1/640 s; EXILIM EX-ZR1700, CASIO Corporation, Tokyo, Japan) captured images of the participant’s entire body from the touchdown of the take-off foot to the landing foot release, from both sides of the hurdle, diagonally forward and backward to the right, and diagonally forward and backward to the left. Before the trial, coloured plastic markers were fixed on the ground as control points every 3 m (3 points) in the direction of the sprint and every 1.2 m (2 points) to the side. Moreover, a 2.05-m calibration pole with control points at 0.5-m intervals (5 points) was moved to the position of each coloured marker and placed vertically on the ground. All control points were captured by their respective cameras (30 control points in total: 3 in the direction of sprint × 2 in the lateral direction × 5 in the vertical direction). The respective minimum and maximum standard errors for calculating the 3D coordinate values of the body analysis points were 0.003–0.007 m, 0.004–0.008 m, and 0.008–0.010 m. This accuracy was sufficient for the construction of 3D coordinates in this study.

### Design and Procedures

Before the trials, participants warmed up using dynamic stretching with hurdles, sprint drills, and three 50-m sprints, and practised hurdles at least three times in the trial setting. Each participant was instructed to take off at step 9 from the starting position. The starting position was moved within a range of 10–50 cm to limit the approach distance, forcing serious stride adjustment so that the athlete could transition from a smooth sprinting motion to the take-off. If any body part contacted the hurdle and the hurdle collapsed, if the take-off or landing was disrupted, or if there was an obviously large deceleration, the trial was considered invalid. Moreover, participants were instructed to keep their form and effort unchanged until the 30-m point so that their sprinting behaviour would not change substantially after landing. Hurdle clearance was measured twice for each participant. All participants completed no more than four measurement attempts.

The experimental setups and procedures were designed to remove the influence of height and interval limitations on hurdle clearance. The specific reasons were as follows: (1) Partial correlation analysis controlling for height cannot completely exclude the effects of height differences on study results (Nagahara et al., 2021). Generally, clearing a hurdle that is lower relatively to height results in less deceleration (Ozaki and Ueda, 2022a). Therefore, a hurdler may have a large take-off deceleration due to short height, but excellent free-limb technique. Because the purpose of this study was to investigate efficient free-limb techniques related to good take-off variables, internal validity was a priority. Therefore, in this study, a hurdle of the same height as the CM height at the anatomical upright position of each participant was used. (2) Using the men’s 110 m hurdles setting according to official race regulations, the approach stride strategy produces large differences in spatiotemporal variables of hurdle clearance (Rowley et al., 2021). (3) Hurdle clearance with full-effort acceleration from the block start was unfamiliar to the 400 m hurdlers. (4) At longer approach distances, differences in stride adjustment ability can affect the take-off technique owing to increased stride accumulation error (Ozaki et al., 2019). For these reasons, the approach distance was set to 15 m, an arbitrary distance at which all participants were able to run with the same number of steps (9 steps) and without noticeable adjustments in posture or step length during pretest measurements conducted with two 110 m and two 400 m hurdle specialized competitors, respectively. Although the hurdle clearance measured in this experimental setting corresponded to around the first hurdle of 110 m hurdles, it was a reasonable setting to exclude the effects of interval limitations, height, and ability to adjust stride and to properly assess individual hurdle clearance techniques (Ozaki and Ueda, 2022a). In addition, participants in this study were of shorter height than top-level hurdlers (1.87 ± 0.05 m) (Hanley et al., 2021). Therefore, rather than using a competition hurdle setting, a relative height hurdle was used to ensure external validity. The horizontal distance between the toe of the support leg and the hurdle at the take-off and one step before the take-off, and the length of the preparatory step just before the take-off were 1.89 ± 0.16 m, 3.47 ± 0.18 m and 1.58 ± 0.08 m, respectively. The standard deviations in the two measured trials were 0.03 ± 0.02 m, 0.04 ± 0.03 m and 0.05 ± 0.02 m, respectively. This variability was markedly lower than that reported in previous studies (Ozaki et al., 2019; Panteli et al., 2023; Smirniotou et al., 2022). The intraclass correlations were as high as 0.920, 0.868, and 0.721, respectively. Therefore, it was determined that in the setup of this study, hurdlers completed the take-off in the ideal position, with no noticeable step length adjustments immediately prior to the take-off. Furthermore, the results obtained in such a setting have the advantage of being fundamental and can be adapted to the take-off techniques of various hurdle events.

### Data Analysis

Video images were captured on a personal computer, and a skilled examiner manually digitized 28 body feature points at 120 Hz using a video motion analysis system (Frame-DIAS V, DKH Retail Limited, Cheltenham, United Kingdom). The joint centres of the elbow, ankle, and knee joints were the midpoints of the markers placed on the lateral and medial sides of each joint. The left and right hip joint centres were estimated using the method proposed by Kurabayashi et al. (2003). Other joint centres were digitized directly using markers affixed to the body surface as guides. The CM heights of participants were calculated using a 2D direct linear transformation method (Walton, 1979) based on calibration. The CM coordinates were obtained using the inertial parameters of the 14-segment model reported by Ae (1996). All trials were digitized from 10 frames before touchdown for the take-off until 10 frames after the toe release. Based on these data, the real coordinates of each trial were obtained using the 3D direct linear transformation method (Shapiro, 1978). The obtained 3D coordinates were smoothed for each coordinate component of each analysis point using a Butterworth digital filter after determining the optimal cut-off frequency using the residual analysis method (Wells and Winter, 1980). The actual cut-off frequencies were in the ranges of 6–13 Hz, 6–12 Hz, and 7–13 Hz in the running, lateral, and vertical directions, respectively. The coordinate data were projected onto the sagittal plane and analysed in a 2D plane.

Digitization reliability in calculated variables was confirmed by intraclass correlation coefficients, with the same examiner digitizing the same test twice, separated by at least 48 h (Paradisis and Cooke, 2001; Paradisis et al., 2019). These coefficients ranged from 0.712 to 0.999, and 15 of the 23 coefficients were above 0.9, indicating good intrarater reliability.

### Variables

The mean of the two trials was used to calculate the variables. The variables used in this study were as follows:

### Take-off Variables

- **Take-off velocity (m/s)**: Horizontal velocity of the CM in the direction of running in the frame in which the support leg toe was released from the ground. The moment of touchdown and the toe off was determined by the maximum vertical acceleration of the toe (Nagahara and Zushi, 2013). For heel-contact hurdlers (n = 2), the touchdown moment was only visually determined. The intrarater reliability of the touchdown moment in heel-contact participants was performed by a visual re-rating by the same examiner at an interval of at least 48 hours. The inter-rater reliability was determined visually by two examiners. The touchdown moments in heel contact participants were all consistent by both reliability measures.
- **Velocity change rate (%)**: Change rate of horizontal velocity of the CM in the running direction from one frame before the toe touchdown to the toe off.
- **Support time (s)**: The time from the touchdown to the toe off.

### Vertical Momentum Variables

**Figure 1:**
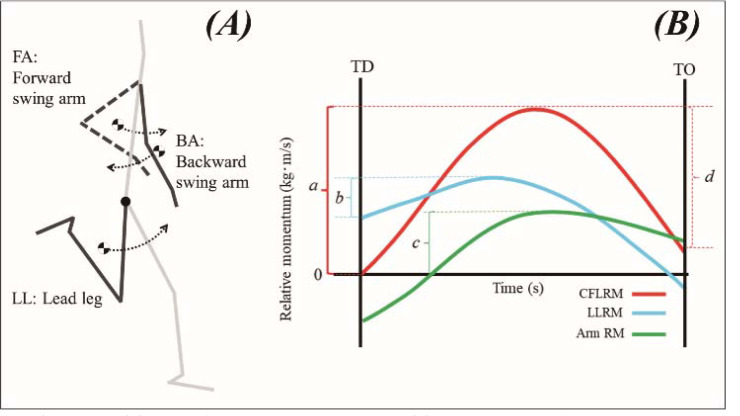
Definition of free limbs (A) and overview of free limb contribution using the relative vertical momentum method (B). *CFLRM: Sum of vertical momentum to the proximal joints of the lead leg and right and left arms. LLRM: Relative vertical momentum to the proximal joint of the lead leg. Arm RM: Sum of the relative vertical momentum of the left and right arms relative to the proximal joint*.

Vertical momentum variables were calculated based on Lees and Barton (1996) and Lees et al. (2000). Figure 1 shows the definition of free limbs and the calculation of the contribution of free limbs using the RM method in this study. Based on Lees et al. (2000), who analysed the relative vertical momentum of the free limb in the high jump, the authors created a brief outline of the contribution of the free limb to the vertical velocity (Figure 1A). To simplify the illustration, the relative vertical momentum of the arms is shown only as the sum of the left and right arms. a: Sum of the relative vertical momentum positive increase for all free limbs. The ratio of (a) to the total body vertical momentum at the toe off is the free limb's contribution to the whole-body vertical momentum. b: The relative vertical momentum positive increase in the lead leg. c: The relative vertical momentum positive increase of the sum of the left and right arms. Negative relative vertical momentum is ignored because it does not contribute directly to a positive increase in whole-body vertical momentum. d: The decrease in total relative vertical momentum of the free limb over the toe off. This is the amount of vertical momentum transmitted to body parts other than the free limb. These interpretations are similar to those described by Lees and Barton (1996) and Lees et al. (2000).

- **RM of the upper limb (kg∙m/s)**
  The CM of the upper limb was defined as the composite CM of the upper arm, forearm, and hand. The coordinates of the upper margin of the sternum were subtracted from the CM of the upper limbs. The RM of the upper limb was defined as the value obtained by the vertical velocity of the CM of the upper limbs relative to the upper margin of the sternum, multiplied by the mass of the upper limb. The RM of the upper limb was calculated for the forward swing arm (FA) and the backward swing arm (BA).
- **RM of the lead leg (kg∙m/s)**
  The CM of the lead leg (LL) was defined as the composite CM of the thigh, the shank, and the foot. The coordinates of the centre of the greater trochanters were subtracted from the CM of the LL. The RM of the LL was defined as the value obtained by the vertical velocity of the CM of the LL relative to the greater trochanters, multiplied by the mass of the LL. Thereafter, the RM of each free limb was denoted as LLRM, FARM, and BARM.
- **Combined free limb relative vertical momentum (CFLRM) (kg∙m/s)**
  The CFLRM was defined as the sum of LLRM, FARM, and BARM. Because the negative RM of one limb can cancel out the positive RM of the other limb, CFLRM was used to calculate the overall free limb contribution to WBVM_TO_.
- **RM positive increase (kg∙m/s)**
  The RM positive increase was defined as the increase from the positive minimum to the positive maximum of the RM of each limb. Since negative RM does not directly contribute to an increase in WBVM_TO_, the lowest RM value for each limb was calculated as zero if the lowest RM value was negative.
- **Limb potential (kg∙m/s)**
  The limb potential was defined as the sum of peak LLRM, peak FARM, and peak BARM. This represents the theoretical maximum value of CFLRM that can be generated when all free limbs reach peak RM at the same time.
- **WBVMTO (kg∙m/s)**
  The WBVM_TO_ was defined as the product of the vertical velocity of the CM and the mass of the whole body at the toe off.
- **Limb contribution (%)**
  The limb contribution was defined as the ratio of the LLRM positive increase, the FARM positive increase, the BARM positive increase, and the CFLRM positive increase to WBVM_TO_. This is the index of the free limb’s ratio of contribution to the whole-body vertical velocity.
- **Limb effectiveness (%)**
  The limb effectiveness was defined as the ratio of the CFLRM positive increase to limb potential. This is an indicator of how the free limb was coordinated to maximize its contribution. When limb effectiveness was maximized, the peak LLRM, peak FARM, and peak BARM were reached simultaneously.

### Statistical Analysis

The Kolmogorov-Smirnov test was performed to confirm the normality of the data. Because the velocity change rate was not normally distributed in the data, Spearman’s rank correlation analysis was used for the relationships between the velocity change rate and the other variables. Pearson’s correlation analysis was used for all other normally distributed variable relationships. Data are expressed as the mean ± SD. Statistical analyses were performed using statistical processing software (IBM SPSS Statistics v20.0, IBM, USA). The level of significance was set at *p* < 0.05.

## Results

The take-off velocity was 6.71 ± 0.51 m/s. The velocity change rate was −9.79 ± 3.36%, and all participants decelerated at the take-off. The support time was 0.131 ± 0.014 s. There were significant correlations between the take-off velocity and the velocity change rate (rS = 0.844, *p*=0.0003), the take-off velocity and support time (r = −0.594, *p* = 0.032), and the velocity change rate and support time (rS = −0.737, *p* = 0.004).

**Table 1:** Relationship between take-off variables and RM at each free limb in hurdle clearance.

Variables	Mean ± SD	Take-off velocity	Velocity change rate	Support time
CFLRM at touch down (kg·m/s)	5.02 ± 5.93	0.389	0.418	−0.486
Peak CFLRM (kg·m/s)	22.04 ± 3.66	−0.357	−0.511	0.465
CFLRM positive increase (kg·m/s)	16.31 ± 6.83	−0.288	−0.615*	0.521
LLRM at touch down (kg·m/s)	8.74 ± 2.81	0.451	0.374	−0.413
Peak LLRM (kg·m/s)	15.17 ± 2.56	−0.478	−0.478	0.363
LLRM positive increase (kg·m/s)	6.43 ± 3.69	−0.675*	−0.621*	0.567*
FARM at touch down (kg·m/s)	−1.25 ± 2.17	0.253	0.582*	−0.457
Peak FARM (kg·m/s)	4.42 ± 1.51	−0.353	−0.478	0.463
FARM positive increase (kg·m/s)	4.02 ± 2.05	−0.272	−0.621*	0.519
BARM at touch down (kg·m/s)	−2.47 ± 2.04	0.240	0.225	−0.356
Peak BARM (kg·m/s)	4.31 ± 1.71	0.048	−0.121	0.170
BARM positive increase (kg·m/s)	4.31 ± 1.71	0.048	−0.121	0.170
Limbs potential (kg·m/s)	23.9 ± 3.87	−0.432	−0.445	0.495
WBVM_TO_ (kg·m/s)	150.21 ± 20.69	−0.711**	−0.813**	0.632*
Limbs contribution of combined free limb (%)	10.72 ± 4.08	0.003	−0.242	0.336
Limbs contribution of LL (%)	4.14 ± 1.90	−0.539	−0.500	0.490
Limbs contribution of FA (%)	2.61 ± 1.19	−0.054	−0.467	0.358
Limbs contribution of BA (%)	2.86 ± 1.03	0.322	0.071	−0.052
Limbs effectiveness (%)	66.77 ± 23.06	−0.092	−0.429	0.417
CFLRM peak point (%)	70.88 ± 25.81	0.189	−0.235	0.098
LLRM peak point (%)	68.00 ± 31.39	−0.119	−0.094	0.431
FARM peak point (%)	63.15 ± 23.78	0.140	−0.389	0.222
BARM peak point (%)	75.38 ± 16.30	0.700*	0.273	−0.452

RM: Relative vertical momentum. SD: standard deviation. CFLRM: Combined free limb relative vertical momentum. LLRM: Relative vertical momentum of the lead leg. FARM: Relative vertical momentum of the forward swing arm. BARM: Relative vertical momentum of the backward swing arm. WBVM_TO_: Whole-body vertical momentum at toe off. Because the velocity change rate was not normally distributed in the data, Spearman's rank correlation analysis was used for relationships between the velocity change rate and other variables. Pearson's correlation analysis was used for all other relationships.

Bold font indicates significant differences at: * p < 0.05, ** p < 0.01

**Figure 2:**
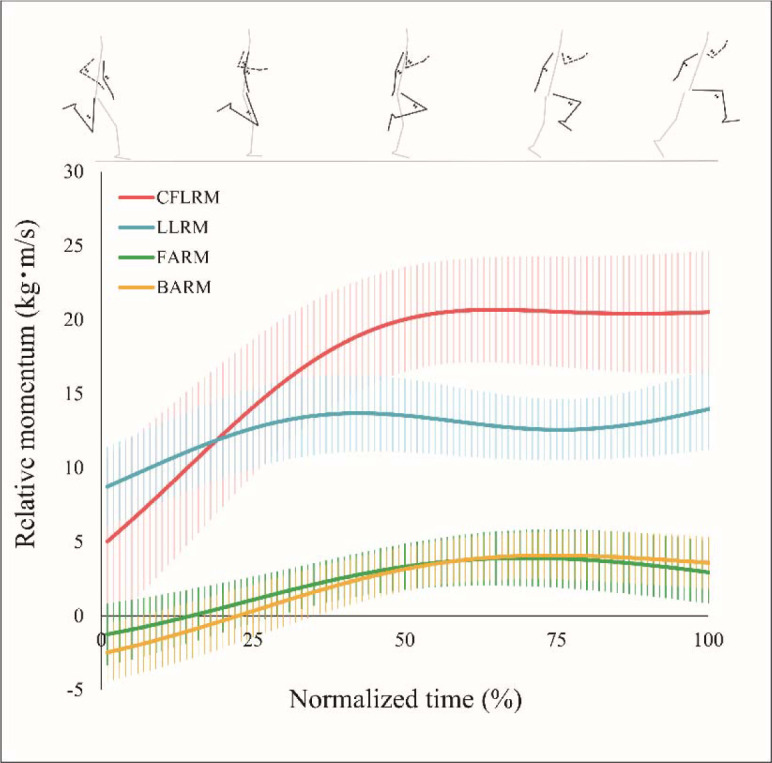
RM change of the free limb at the take-off. *RM: Relative vertical momentum. CFLRM: Combined free limb relative vertical momentum. LLRM: Relative vertical momentum of the lead leg. FARM: Relative vertical momentum of the forward swing arm. BARM: Relative vertical momentum of the backward swing arm*.

Figure 2 shows the overall average RM changes of the free limb at the take-off. The CFLRM increased until the mid-support phase and was then maintained until release. The LLRM increased toward the middle of the support phase, then decreased, and then increased again toward the release. The FARM and BARM increased until the late support phase and showed a gradual decrease toward the release.

## Discussion

The results of this study showed that hurdlers with shorter support time, smaller deceleration, and higher take-off velocity had lower WBVM_TO_. Higher CFLRM, LLRM, and FARM positive increases, which contributed to increased WBVM_TO_, were associated with lower performance at the take-off. Therefore, it was important for the hurdler to reduce the absolute contribution to vertical momentum from the LL and FA and prevent an increase in WBVM_TO_ to improve take-off performance. Based on the above, the hypothesis of this study was partially accepted.

The mean CFLRM reached its peak in the middle of the support phase and was then maintained over the take-off. This indicates that the vertical momentum of the free limb was minimally transferred to the rest of the body (trunk and support leg) compared to that in other jumping tasks (Lees and Barton, 1996; Lee et al., 2000). This reduced the increase in the trunk CM and hurdle clearance with a low whole-body CM. Since FARM and BARM decreased toward the toe off, the reason why CFLRM was maintained until the toe off was an increase in LLRM. Hurdle clearance take-off requires the knee of the LL to be pulled up high until it crosses the hurdle (Bedini, 2016; McDonald, 2002). Therefore, this increase in LLRM in the latter half of the support phase may be due to the swing direction of the LL coinciding with the vertical direction. Thus, the hurdle clearance take-off showed specific RM changes in the free limb due to the unique jumping style of hurdle clearance relative to other jumping and running exercises (Lees and Barton, 1996; Lee et al., 2000).

Lees and Barton (1996) and Lees et al. (2000) pointed out the importance of synchronizing free limb RM peaks to enhance jumping performance. In coaching hurdle clearance, dynamic and specific upper limb and LL movements are required compared to sprinting (Bedini, 2016; McDonald, 2002; McDonald and Dapena, 1991a). Under these premises, the results of this study suggest the importance of coordination in hurdle running rather than avoiding synchronization of the peak RM of the free limbs. In particular, since the BARM peak point showed a significant positive correlation with take-off velocity, it may be important to delay the vertical pull-up of the BA.

Furthermore, the correlation analysis of this study showed that hurdlers with shorter support time, smaller deceleration, and higher take-off velocity maintained a higher FARM already at the touchdown, preventing an increase in the RM during the support phase. In this regard, a higher CM height at the take-off in hurdle clearance is associated with a faster sprint velocity in hurdle running (Čoh, 2003; Mauroy et al., 2014; Ozaki and Ueda, 2022b). Furthermore, it is effective to move the FA forward early during the take-off to compensate for the angular momentum of the horizontal axis for the trail leg forward movement toward the landing (Bedini, 2016; McDonald and Dapena, 1991a). The outward swing of the FA at the take-off compensates for the angular momentum of the horizontal axis due to the forward swing of the LL and increases LL swing speed. This shoulder joint abduction in preparation for the outward swing increases FARM at the touchdown and contributes to minimizing the RM positive increase during the support phase. Therefore, swinging the FA to approach the upper margin of the sternum at touchdown, rather than swinging downward at the shoulder joint, might be a reasonable strategy to compensate for LL and trail leg motion.

The LLRM positive increase was the only free limb RM that showed significant correlations with all take-off variables. To suppress this LLRM positive increase, it is a reasonable strategy to increase the LLRM at the touchdown. For high LLRM at the touchdown, the LL should have a high vertical velocity at the touchdown as the LL swings more toward the CM of the whole body. This technique minimizes braking time by closing the horizontal distance between the touchdown toe position and the CM of the whole body (Hunter et al., 2005). In fact, this short horizontal distance between the toe and the whole-body CM at the touchdown is a spatiotemporal variable strongly associated with deceleration in the hurdle take-off (Ozaki and Ueda, 2022b). In addition, hurdlers shorten the preparation step length before the take-off and suppress the backward movement of the LL to swing the LL forward early (Nagahara et al., 2021; Mauroy et al., 2014). Furthermore, the high swing-up of the LL thigh at the toe-off is a key factor for hurdle clearance without hitting the hurdle (Iwasaki et al., 2022). The psoas major on the hurdler's LL side is more hypertrophied than on the trail side, which may be associated with an earlier forward swing of the LL in superior hurdlers (Okutani et al., 2017). Thus, there is much evidence regarding the benefit of hurdlers swinging their LL forward early and achieving a high LLRM at the touchdown. However, LLRM at the touchdown was not significantly correlated with the take-off variable in this study. Therefore, there may be other techniques useful to suppress the positive increase of LLRM. One of these is to increase LL knee flexion at the touchdown. Knee flexion raises the CM height of the LL at the touchdown and reduces the vertical displacement of the LL during the swing by shortening the radius of rotation of the LL around the greater trochanter. This shortens the distance that the LL can accelerate vertically during the support phase and contributes to the reduction in the positive increase of LLRM. This point needs to be clarified in further studies, including lower limb joint angles and joint angular velocities.

In light of the above, coaches must understand that the free-limb movements required for hurdle clearing are fundamentally different from those required for jump events, where the emphasis is on vertical velocity acquisition. Therefore, hurdlers should emphasize hurdle-specific drills, including quick LL and FA swings, in addition to hurdle running in a competition setting. In doing so, efforts should be made to keep the LL and FA close to the torso and high at the moment of the touchdown. A short preparation step should also be emphasized as it will assist in the proper positioning of these free limbs. In addition, from the touchdown to the take-off, it is necessary to hold LL knee flexion and suppress any increase in vertical momentum.

## Limitations

The use of manually digitized video has accuracy limitations compared to more preferred methods like optical 3D motion capture. Moreover, take-off in hurdle clearance is affected by differences in interval length and hurdle height due to differences in sex, events, heights, and record levels (González-Frutos et al., 2019; Nagahara et al., 2021; Ozaki and Ueda, 2022a; Panoutsakopoulos et al., 2020). For example, a hurdler with low running velocity and small step length may utilize mechanics that intentionally achieve a large vertical impulse with a long support time and a large vertical CM velocity to shorten the running distance of the interval. The take-off velocity of the participants in this study (6.71 ± 0.51 m/s) was lower than the hurdle step velocity of elite hurdlers (8.57 ± 0.23 m/s) (McDonald and Dapena, 1991b) and sub-elite hurdlers (7.42 ± 0.24 m/s) (Amara et al., 2019), and was considered to be typical of the 110 m hurdles at these lower performance levels. Therefore, when implementing the study results into practice, these event characteristics and the individuality of the hurdler must be considered. Additionally, the kinematics of the first hurdle clearance in the acceleration phase and the subsequent hurdle clearance are different (González-Frutos et al., 2020). The hurdle heights used in this study were relative to the CM height in the anatomical upright position. Therefore, different sprinting velocities, competition levels, absolute hurdle heights, and positions of the hurdles analysed could lead to different results in the study. In the future, the range of analysis should be expanded to include the effects of different hurdle heights, preparation steps, and landing steps and to investigate their relationships with the actual ground reaction force.

## Conclusions

This study investigated the relationship between free limb relative vertical momentum dynamics during hurdle take-off and take-off variables in a setting independent of the approach distance, interval distance, and individual height. The results showed that during the take-off, the smaller the increase in relative vertical momentum of the lead leg, the higher the take-off velocity, the shorter the support time, and the smaller the deceleration. In addition, the higher the relative vertical momentum of the forward swing arm at the touchdown and the smaller the increase in relative vertical momentum of the forward swing arm and combined free limbs during the take-off, the smaller the deceleration during the take-off. This result may also be related to the outward swing with shoulder joint abduction in the forward swing arm. Therefore, the hurdler must minimize deceleration at the take-off by pulling the lead leg and the forward swing arm high and forward at the touchdown with a high swing speed to reduce the increase in vertical velocity of the free limbs during the take-off.
